# Supplementary material for: The AP2/ERF GmERF113 Positively Regulates the Drought Response by Activating GmPR10-1 in Soybean
Source: Int J Mol Sci. 2022 Jul 24;23(15):8159. doi: 10.3390/ijms23158159 (PMC9330420; doi:10.3390/ijms23158159)
Supplement: Supplementary file 1 [file ijms-23-08159-s001.zip › Supplementary File 1.pdf]

Supplementary Information: Overview

The following supplementary data are available for this article in this document:

Figures

- **Figure S1** Identification of the *GmERF113* transgenic soybean plants used in this study
- **Figure S2** Identification of the *GmPR10-1* transgenic soybean hairy roots used in this study

Tables

- **Table S1** List of primers used in this study
- **Table S2** DEGs associated with drought stress response from *GmERF113*-OE RNA-sequencing (Fold Change >1.4)

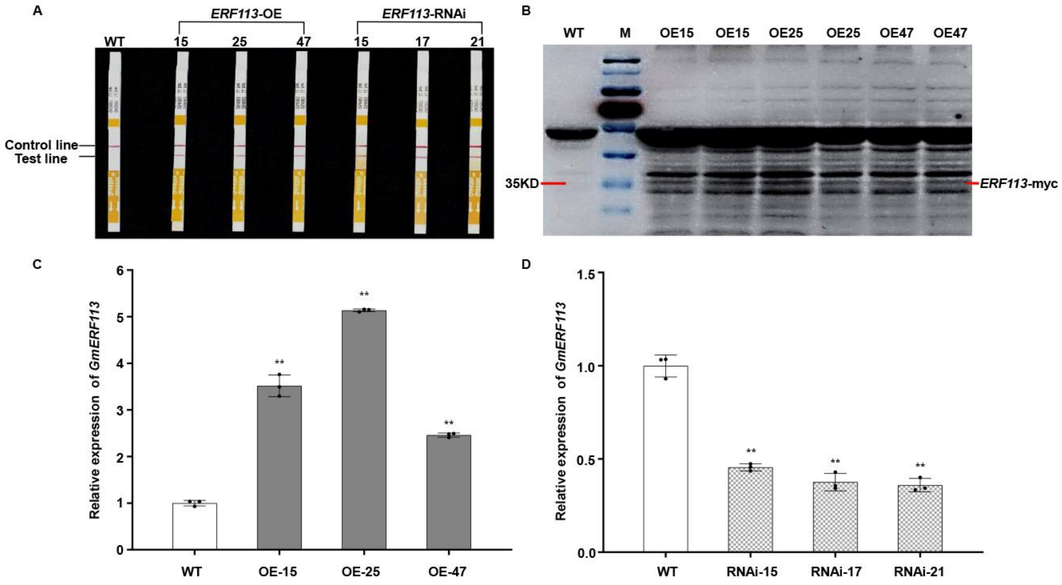

Figure S1. Identification of the *GmERF113* transgenic soybean plants used in this study. **(A)** The three independent T_3 *GmERF113-OE*, three independent T_3 *GmERF113-RNAi* and wild-type (WT) soybean plants were tested using QuickStix Kit for LibertyLink (bar) strips. **(B)** Immunoblots showing the expression of the GmERF113-Myc fusion protein in T_3 *GmERF113-OE* soybean plants and the WT controls. The total protein extracts were analyzed using a 12% SDS-PAGE, and the immunoblot was probed with anti-Myc antibody. **(C)** Relative expression level of *GmERF113* in the three independent T_3 *GmERF113-OE* plants and WT controls. **(D)** Relative expression level of *GmERF113* in the three independent T_3 *GmERF113-RNAi* plants and WT controls. The reference soybean gene *GmActin4* and *GmTubulin4* were used as internal control to normalize the data. The experiments were performed on three biological replicates, each with three technical replicates, and were statistically analyzed using Student's *t*-test (**$P < 0.01$). Bars indicate the standard deviation of the mean.

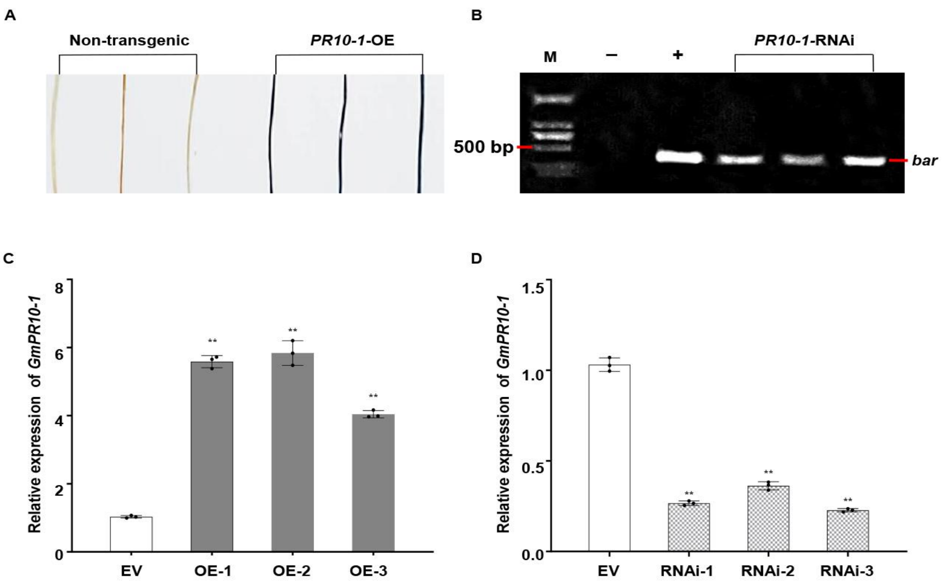

Figure S2. Identification of the *GmPR10-1* transgenic soybean hairy roots used in this study. **(A)** GUS staining of the *GmPR10-1-OE* transgenic hairy roots and the non-transgenic controls. **(B)** *GmPR10-1-RNAi* transgenic soybean hairy roots examined using PCR of *bar* gene. **(C)** Relative expression level of *GmPR10-1* in the in the *GmPR10-1*-OE transgenic soybean hairy roots and the empty vector (EV) controls. **(D)** Relative expression level of *GmPR10-1* in the *GmPR10-1*-RNAi transgenic soybean hairy roots and EV controls. The reference soybean gene *GmActin4* and *GmTubulin4* were used as internal control to normalize the data. The experiments were performed on three biological replicates, each with three technical replicates, and were statistically analyzed using Student's *t*-test (**$P < 0.01$). Bars indicate the standard deviation of the mean.

Table S1. List of primers used in this study

Primer name	Primers (5'-3')
GmERF113-qF	GATAGCACCCTTTCTTCACCAA
GmERF113-qR	ATGTCTTTTTCTCCCATTTCCT
GmActin 4-F	GATCTACCATGTTCCCAAGT
GmActin 4-R	ATAGAGCCACCAATCCAGAC
GmERF113-MycF	CGGGGGACTCTTGACCATGGTTATGCATCCAATCAATACTG GT
GmERF113-MycR	CCCACGTGGCTAGCAGATCTATTCTTGTCATGAAAATCACT CC
GmERF113-RNAi1F	TTTCATTTGGAGAGGACACGCTCGAGCCAATCAATACTGGT GATGATAGC
GmERF113-RNAi1R	AATCATCGATTGGGCGCGCCCCATGGGAAGAAGAACATGTT CAGGGAAA
GmERF113-RNAi2F	TCCCGGGTCTTAATTAACTCTCTAGACCAATCAATACTGGTG ATGATAGC
GmERF113-RNAi2R	GTCAATTTGCAGGTATTTGGATCCGAAGAAGAACATGTTCA GGGAAA
pGmPR10-1-ChIPF	AGGGGTAGAGGGTGTCACAAAT
pGmPR10-1-ChIPR	CACATGGGCGGCTATTGG
*pGmPR10-1*F	TCGAGGTCGACGGTATCGATAAGCTTAGAGATGTTACTATC GCTGGC
*pGmPR10-1*R	GCGGCCGCTCTAGAACTAGTGGATCCAAGACTTTCAATGTT AGTGGCT
GmPR10-1-F	CCATAGGATCCAACTTTCACAA
GmPR10-1-R	TCCAGCAGGTAGGCTTCCAC
GmPR10-1-GUSF	CTCGAGATGGATTACAAGGATGACGACGATAAGATGGGAG CCACAACTTTTACA
GmPR10-1-GUSR:	CTCGAGTTAAGCATAAACTTGTGGATT
GmPR10-1-RNAi1F	TTTCATTTGGAGAGGACACGCTCGAGATGGGAGCCACAAC TTTTAC
GmPR10-1-RNAi1R	AATCATCGATTGGGCGCGCCCCATGGAGCTATAGACTCAAG CTTGTCC
GmPR10-1-RNAi2F	TCCCGGGTCTTAATTAACTCTCTAGAATGGGAGCCACAACT TTTAC
GmPR10-1-RNAi2R	GTCAATTTGCAGGTATTTGGATCCAGCTATAGACTCAAGCTT GTCC
GmABA8'-OH3-qF	TCTTGCTTGATTGGTGTGTCT
GmABA8'-OH3-qR	AATTCGTCTGTGTTCCTCCTCA
GmPP2C37-qF	TCCAAAGTAGACTACGACGACGA
GmPP2C37-qR	ACACATAGTCGCAACATGAGAGC
GmRGLG1-qF	GCACGCTACAGAGAGATTGGT
GmRGLG1-qR	TTCACCCACCATCCAAGCAA

Table S2. DEGs associated with drought stress response from *GmERF113*-OE RNA-sequencing (Fold Change >1.4)

Gene ID	Gene Name	Regulated	Fold Change
Glyma.03G137900	Galactinol-sucrose galactosyltransferase 2-like	Up	1.4901528625
Glyma.03G101000	ABC transporter C family member 9-like	Up	1.4922739936
Glyma.08G193500	Beta-galactosidase 1-like	Up	1.5407468686
Glyma.06G235500	Tyrosine aminotransferase	Up	1.6781479584
Glyma.12G058300	UDP-glycosyltransferase 76E11-like	Up	2.5548994258
Glyma.04G194800	Calcium-binding protein CML38-like	Up	2.4621104231
Glyma.15G128800	Peroxidase 15-like	Up	2.8285797737
Glyma.08G031400	Zinc finger CCCH domain-containing protein 20-like	Up	1.6439866439
Glyma.16G110700	ABA 8'-hydroxylase 3	Down	5.3125463029
Glyma.18G035000	Protein phosphatase 2C 37-like	Up	1.8748320842
Glyma.06G155100	E3 ubiquitin-protein ligase RGLG1-like	Up	2.3278835777
