# Supplementary material for: The AP2/ERF GmERF113 Positively Regulates the Drought Response by Activating GmPR10-1 in Soybean
Source: Int J Mol Sci. 2022 Jul 24;23(15):8159. doi: 10.3390/ijms23158159 (PMC9330420; doi:10.3390/ijms23158159)
Supplement: Supplementary file 1 [file ijms-23-08159-s001.zip › Supplementary File 2.pdf]

Raw images of the western blot and electrophoretic gel.

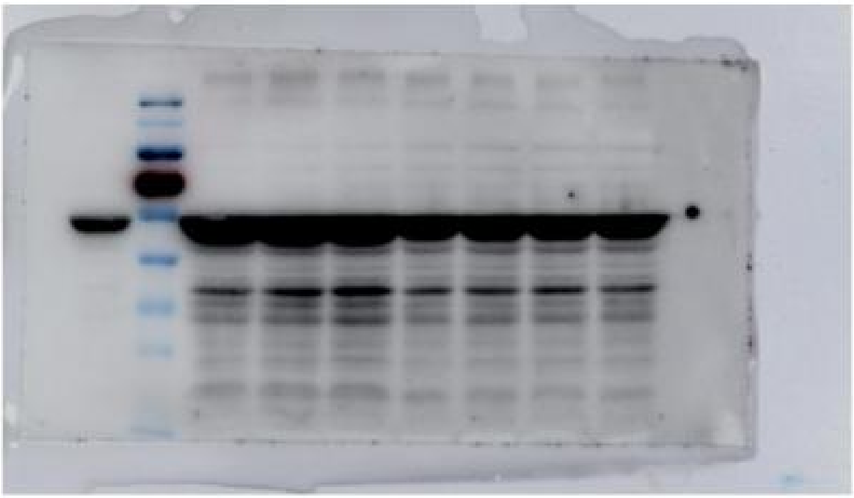

Figure S1B (Raw image): Western Blot image of identification of *GmERF113* -OE transgenic soybean plants

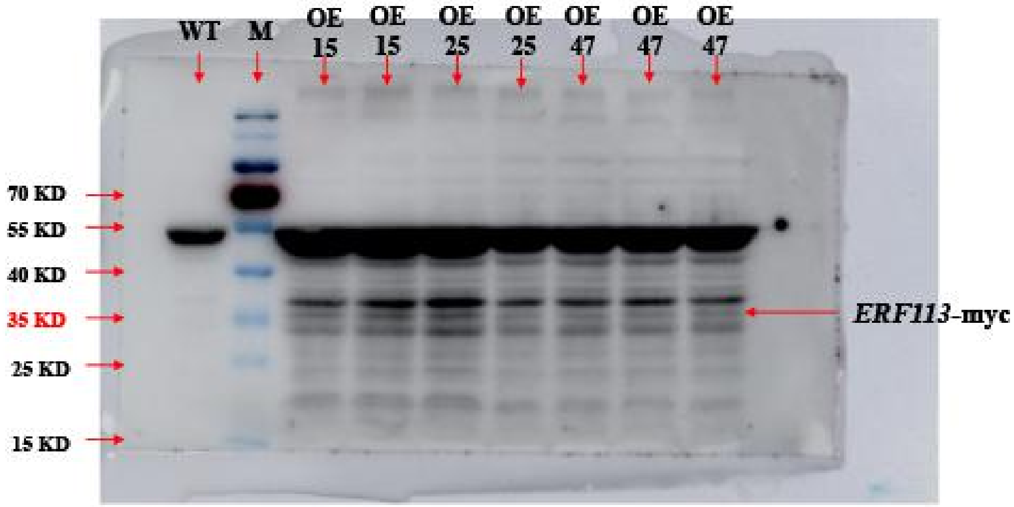

M: Marker; WT: Wide type plant; OE15/25/47: Overexpression of *GmERF113* transgenic soybean plants15/25/47

Figure S1B (Labeled raw image): Western Blot image of identification of *GmERF113* -OE transgenic soybean plants

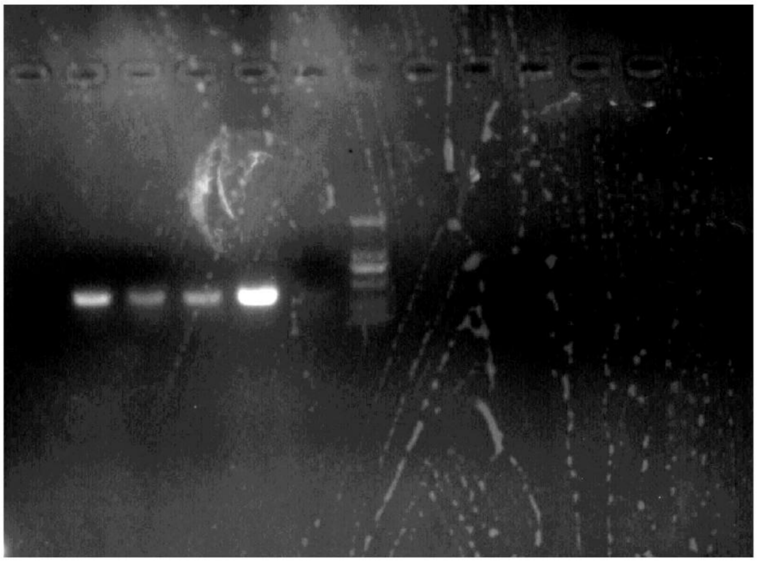

Figure S2B (Raw image): Electrophoretic gel image of identification of *GmPR10-1*-RNAi transgenic soybean hairy roots

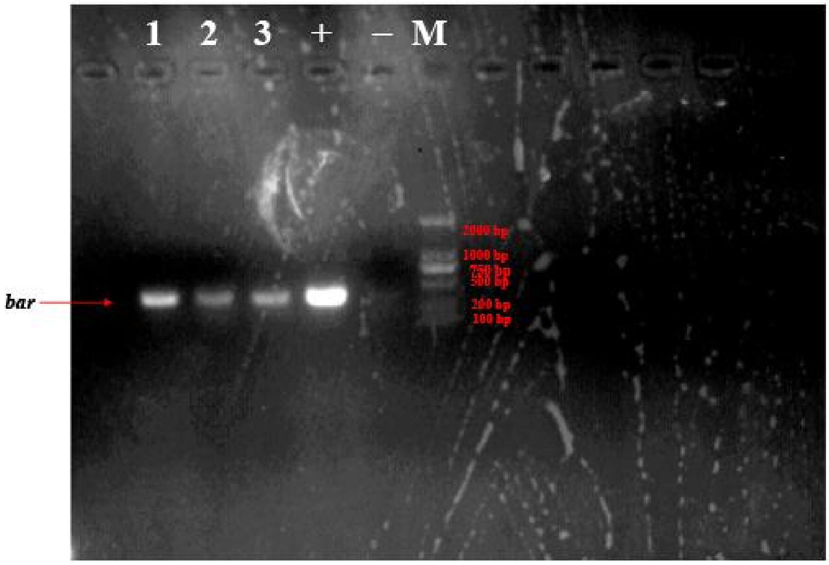

1-3: *PR10-1*-RNAi transgenic soybean hairy roots1/2/3; +: positive control; -: negative control; M: Marker

Figure S2B (Labeled raw image): Electrophoretic gel image of identification of *GmPR10-1*-RNAi transgenic soybean hairy roots
